# Complete non-puerperal uterine inversion caused by uterine hemangioma: about a case report

**DOI:** 10.11604/pamj.2022.42.156.35583

**Published:** 2022-06-27

**Authors:** Skander Abid, Ghassen Ben Dhaou, Ghada Abdelmoula, Ahmed Ben Smida, Mohamed Raouf Ben Abdesslem, Ons Mrad, Mouna Derouiche, Latifa Lassoued

**Affiliations:** 1 University of Sousse, Faculty of Medicine of Sousse, 4000, Sousse, Tunisia,; 2 Farhat Hached University Hospital of Sousse, Department of Obstetric Gynecology, 4000, Sousse, Tunisia

**Keywords:** Non-puerperal uterine inversion, surgical interventions, angioleiomyoma, case report

## Abstract

Uterine inversion is a rare postpartum complication. It is a rare condition in which the internal surface of the uterus protrudes through the vagina. Non-puerperal uterine inversion (NPUI) is extremely rare. In most instances, it is linked to uterine tumors. Among these tumors, leiomyoma is the most frequent cause reported in data. This condition may not be noticed until time of surgery. Malignancy is suspected in most cases. Nevertheless, uterine inversion can be diagnosed preoperatively using radiology. Difficulties in diagnosing NPUI makes this clinical case a challenge in gynaecology and not commonly reported in literature. We report our experience in the diagnosis and treatment of a complete non-puerperal uterine inversion associated with uterine angioleiomyoma. The patient's age was 44, gravida 2 para 1 presented with intermittent vaginal bleeding for four months and an acute abdominal cramping pain. On examination, a large mass lesion was observed which occupies the vaginal cavity and the contour of the uterine cervix could not be reached. Biopsies and Immunohistochemistry matched with an angioleiomyoma. She underwent a transvaginal surgical reposition technique: Spinelli’s. It is important to diagnose accurate non-puerperal uterine inversion. Surgery provides good prognosis and it is necessary. We report a case of NPUI caused by angioleiomyoma. Nevertheless, malignancy must be eliminated in first place.

## Introduction

Puerperal uterine inversion occurs with incidence of 1/2000 delivery and is considered as an emergency. However, NPUI is extremely rare. Most cases of non-puerperal uterine inversion are associated with tumors, mostly leiomyoma. This article reports our experience in the diagnosis and treatment of non-puerperal uterine associated with angioleiomyoma. This later has been reported twice in the literature.

## Patient and observation

**Patient information:** a 44-year-old woman gravida 2 para 1 with a history of intermittent vaginal bleeding presented with an acute abdominal cramping pain. The chronic vaginal bleeding resulted in an anemia (7g/dl). She had no medical, family, psycho-social history and no relevant past interventions.

**Figure 1:**
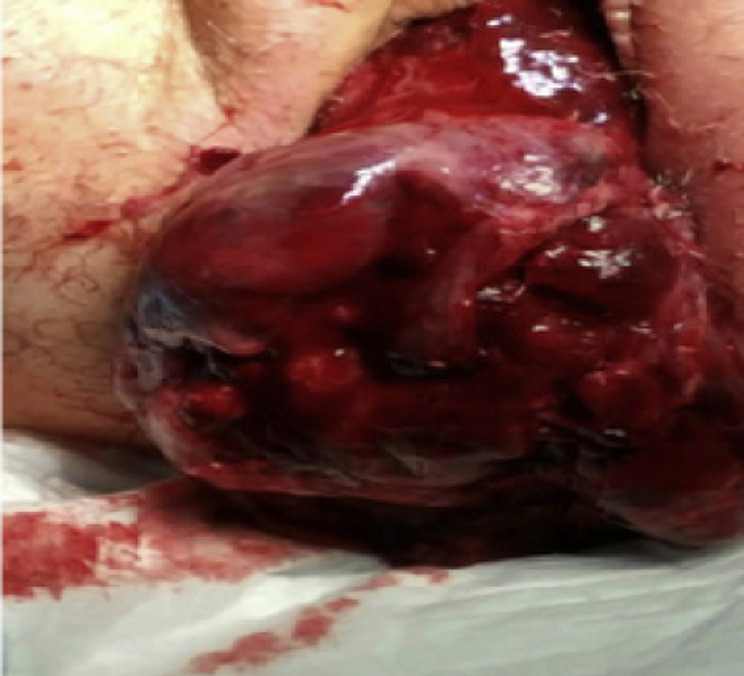
perception of an externalized mass through the vagina related to the inversion of the uterine fundus

**Clinical findings:** on examination; a spontaneously bleeding mass lesion occupying the vaginal cavity was observed hiding the contour of the uterine cervix (Figure 1). On bimanual examination; severe uterine tenderness when mobilizing the cervix was reported. No pelvic mass was found on abdominal palpation.

**Timeline of current episode and diagnostic assessment:** we performed direct biopsies. No malignancy was showed. Immunohistochemistry indicated an angioleiomyoma. Ultrasonographic findings showed a vaginal tissular mass measuring 3.2*4 cm, an hypo echogenic depression and a groove at the uterine fundus.

**Figure 2:**
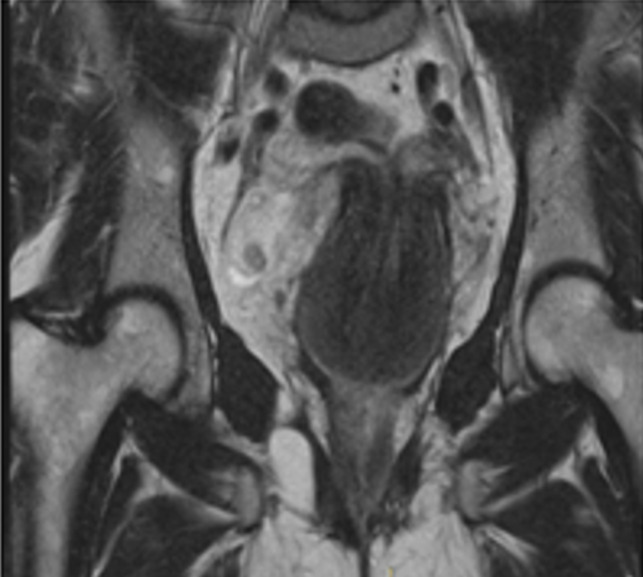
uterine inversion grade 2

**Diagnosis:** uterine inversion grade 2 was diagnosed within MRI (Figure 2) and a surgery was planned.

**Figure 3:**
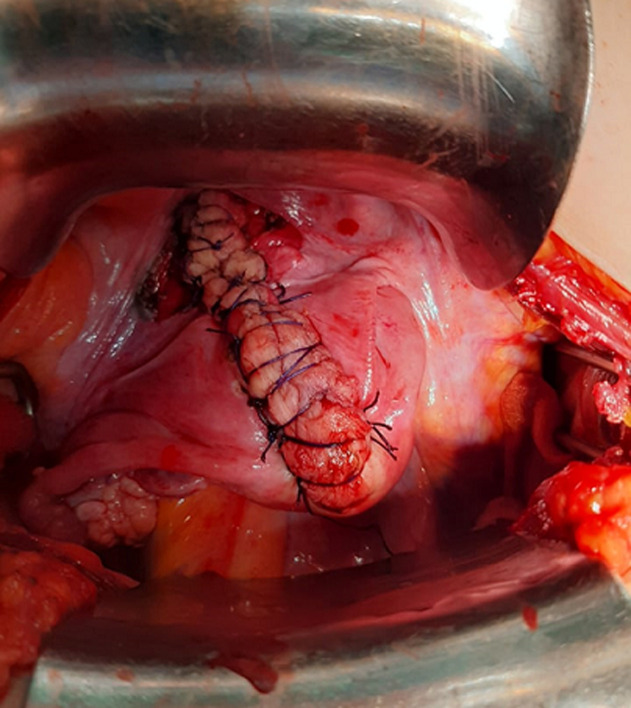
spinelli's approach-dissection of the bladder and anterior uterine wall incision with manual uterine reversion

**Therapeutic interventions:** at laparotomy; central dimpling of the fundus was detected along with a protrusion of the fallopian tubes and ovaries and they were congestive. Spinelli´s approach was adapted; requiring the dissection of the bladder and an anterior uterine wall incision. A manual uterine reversion was realised (Figure 3).

**Follow-up and outcome of interventions:** there was no other intraoperative complication noted. The patient clinical case required a hospitalisation of 8 days before being discharged.

**Informed consent:** written informed consent was obtained from the patient for publication of this case report and any accompanying images.

## Discussion

Uterus inversion is encountered as an obstetric emergency due to postpartum haemorrhage risk. It is extremely rare in non-obstetric instances. To the best of our knowledge, 150 cases were reported from 1987 to 2004 [1]. Das *et al*. reported 47 cases of NPUI related to fibroids (87%), to sarcoma (7.4%) and to carcinoma (5.6%) [2]. Non-puerperal uterine inversion due to Haemangioma was reported only once by Taiwanese Journal of Obstetrics and Gynaecology [2].

**Mechanisms of non-puerperal uterine inversion:** uterine tumors are well-known for weakening and thinning the uterine wall. Concurrent contractions of the uterus results in expelling the tumor through the cervix, thus into the vagina. Angioleiomyoma may also soften the uterine fundus and cause the uterine inversion.

**Clinical appearances in chronic forms:** chronicity is what separates NPUI from obstetrical cases. The presenting symptoms are vaginal bleeding, urinary disturbance and chronic cramping pain of abdominal wall. NPUI can be incomplete, complete or total. In Tunisia, advanced cervical cancer is still diagnosed because cervical smears are not common among women. The clinical appearance of our patient was quite similar to an advanced cervical cancer. Therefore, cervical cancer was predicted after the biopsy. The clinical diagnosis of uterine inversion is challenging and may not be made until the time of surgery. An indentation with a longitudinal hypo echoic groove of the fundal uterus under trans abdominal ultrasound was reported as the main finding suggesting the inversion [3]. Nevertheless, such typical appearance is not specific to uterine inversion and can be absent due to the impact of uterus tumor changing pelvic anatomy. In case of diagnostic difficulties with ultrasound, MRI is still indicated to establish the diagnosis and to also delineate the lesion in neighbor structures [4]. Another case report of a 67-year-old patient who was presented with severe bleeding, mass prolapsed out of the vagina. She underwent a total abdominal hysterectomy with bilateral salpingo-ophorectomy. Pathological evaluation revealed an adenosarcoma of the uterine fundus. Therefore, uterine sarcoma may be associated with uterine inversion. Thus, malignancy should be suspected in non-puerperal uterine inversion. Another case of a 51-year-old multiparous woman whose the initial evaluation suggested an advanced cervical cancer was reported [5]. Nevertheless, clinical examination revealed chronic uterine inversion secondary to fundal sub mucous uterine leiomyoma. A removal of the tumor was done. Histological results confirmed benign uterine leiomyoma. Chronic uterine inversion can be misdiagnosed as advanced cervical cancer. Clinical findings, ultrasound-MRI and histology profile can help distinguish between these two different pathologies with opposite prognosis [6].

**Treatment:** basically, treatment is defined by whether the condition is acute or chronic, the etiology of the inversion and puerperal context. It is recommended to perform a re-inversion before proceeding to hysterectomy. Repositioning of the uterus is usually done after the tumor has been removed and malignancy must be excluded. Unlike acute puerperal uterine inversion in which manual repositioning of the uterus is possible, surgery is imperative in chronic inversion: 1) transvaginal surgical reposition techniques: Spinelli´s approach: first proceed with a dissection of the bladder then anterior uterine wall incision; Kustner´s approach: posterior incision of the uterine wall, sparing the surgeon the risk of lesions of the bladder. 2) Huntington procedure: a vertical incision in the posterior portion of the cervical ring and gentle traction on the round ligaments. 3) Laparoscopic: requests a surgical expertise. Both laparoscopic and vaginal approach can be combined in the treatment of NPUI [7]. 4) Surgical video demonstration of robotic assisted surgery was reported and helped perform a total laparoscopic hysterectomy and bilateral salpingo-oophorectomy in the setting of complete uterine inversion [8].

## Conclusion

Uterine inversion rarely occurs in a non-obstetrical setting. It is essential to exclude a potential malignancy of the uterus as a possible cause of NPUI for proper treatment. This case also suggested that uterine angioleiomyoma might be one of the causes associated with non-puerperal uterine inversion.
